# The effect of feature image on sensitivity of the statistical analysis in the pipeline of a tractography atlas-based analysis

**DOI:** 10.1038/s41598-017-12965-5

**Published:** 2017-10-04

**Authors:** Junya Mu, Qing Xu, Jie Tian, Jixin Liu

**Affiliations:** Center for Brain Imaging, School of Life Science and Technology, Xidian University, Xi’an, 710126 P.R. China

## Abstract

Tractography atlas-based analysis (TABS) is a new diffusion tensor image (DTI) statistical analysis method for detecting and understanding voxel-wise white matter properties along a fiber tract. An important requisite for accurate and sensitive TABS is the availability of a deformation field that is able to register DTI in native space to standard space. Here, three different feature images including the fractional anisotropy (FA) image, T1 weighted image, and the maximum eigenvalue of the Hessian of the FA (hFA) image were used to calculate the deformation fields between individual space and population space. Our results showed that when the FA image was a feature image, the tensor template had the highest consistency with each subject for scalar and vector information. Additionally, to demonstrate the sensitivity and specificity of the TABS method with different feature images, we detected a gender difference along the corpus callosum. A significant difference between the male and female group in diffusion measurement appeared predominantly in the right corpus callosum only when FA was the feature image. Our results demonstrated that the FA image as a feature image was more accurate with respect to the underlying tensor information and had more accurate analysis results with the TABS method.

## Introduction

Clinical neuroimaging studies increasingly rely on diffusion tensor imaging (DTI), which is unique in providing rich information regarding the properties and structure of brain white matter (WM) *in vivo* ^1–4^. With the popularity of DTI, clinical and neuroscience questions related to WM were often addressed by quantitative analysis for regions of specific white matter tracts^5^. Reviewing the number of DTI analyses, the voxel-based analysis (VBA) method is highly sensitive to registration accuracy and tract-based spatial statistics (TBSS) method is limited to skeleton voxels with many WM regions being neglected^6,7^. To address the limitation above, tractography atlas-based analysis (TABS), as an optimized white matter analysis which creates a voxel-wise statistical framework for detecting and understanding white matter differences along a fiber tract, has been used frequently in WM pathway studies^8–10^. In the pipeline of the TABS method, comparison of diffusion properties along a fiber tract requires a method for identifying corresponding anatomical regions^11^. Hence, accurate alignment of the brain anatomy between subjects may potentially affect the accuracy of voxel-wise statistics.

The TABS method contains two major steps, the construction of a diffusion tensor (DT) template and a statistical model for each voxel along the fiber tracts^12,13^. For the template construction, all tensor images in native space are aligned to the population space with a non-linear deformation field^6,14–16^. Integrating spatial and diffusion tensor information in all subjects, a population template is constructed to provide spatial normalization for the analysis of diffusion values at corresponding locations along the fiber tracts^17,18^. In order to minimize the anatomical variability between studied brain structures, Goodlett *et al*.^11^ pointed out that the feature image is a sensitive detector of microstructure in the brain that has crucial importance during template construction^11^. For statistical model building, the fibers in the population space were first mapped into one common coordinate system with a parameterization method, and then the parameterized fibers were warped back into the individual’s native space with an inverse deformation field to collect the diffusion measure^11,13^. Accordingly, the accuracy of the deformation field affected by the selection of the feature image has a significant impact for the matching degree of the corresponding regions in the fibers between the population space and native space. Hence, the selection of the feature image as one of the key steps should be taken into account for the pipeline of the TABS method.

The basic role of a feature image, which should have ultra-sensitivity to identify the corresponding regions of white matter geometry, is transformed into standard space to obtain the deformation field^6,19^. An inappropriate feature image used in registration will lead to inaccurate spatial normalization and inconsistent tract positions^20^. The FA image, as an indirect measure of WM integrity, was first employed to transform individual diffusion weighted images to standard space and obtain the same anatomical structure^6,21–23^. To further improve the sensitivity of the geometry of brain white matter, Goodlett, *et al*.^11^ defined a feature image by calculating the maximum eigenvalue of the Hessian of the FA image (hFA), which was suggested to be a good detector of major fiber bundles with tubular or sheet-like structures^11^. Additionally, considering the high signal to noise ratio and anatomical structure, several researchers also suggested that T1 weighted images could be used to minimize local differences in brain white matter shape across subjects and attain superior results of tensor image alignment^7,24,25^. However, for the three widely used feature images, how or whether the effect of different feature images for spatial alignment between each subject influences the quantitative statistical analysis in the pipeline of the TABS method is still largely unclear.

In the current study, we assumed that the feature image will affect the sensitivity of the statistical analysis in the pipeline of the TABS method by affecting the spatial consistency between each subject. In order to verify the hypothesis, we attempted to construct three study-specific white matter diffusion tensor templates by using different feature images (T1 weighted image, FA image, and hFA image) based on simulation data and experimental data. Subsequently, numbers of similarity metrics were used to evaluate the accuracy and precision of the spatial consistency for all pairs of subjects. Finally, in order to validate the accuracy of the along-tract statistical analysis, we conducted experiments to compare the FA patterns between a male group and a female group of subjects. Based on the difference between the two groups, the receiver operating characteristic (ROC) curve was used to calculate the accuracy of gender classification.

## Materials and Methods

### Subjects

Sixty-six healthy age-, education-, and gender-matched, right-handed Chinese controls were recruited (33 males, age 21–24 years (mean ± SD:22.5 ± 1.5 years); 33 females, age 20–25 years (mean ± SD:22.5 ± 2.5 years)). The exclusion criteria were: (1) macroscopic brain T2-visible lesions on MRI scans, (2) existence of a neurological disease, (3) physical deformities, (4) alcohol, nicotine or drug abuse, and/or (5) claustrophobia. All research procedures were approved by the First Affiliated Hospital of Xi’an Jiao Tong University. Human Studies Subcommittee was conducted in accordance with the Helsinki Declaration. Each participant signed an informed consent respectively.

### Data Acquisition

All subjects were scanned on a 3.0 Tesla GE Excite scanner using an eight channel coil (GE Medical Systems, Milwaukee, WI). DTI images were obtained with a single-shot echo-planar imaging sequence where the diffusion sensitizing gradients were applied along two repeats of 30 non-collinear directions (b = 1000 s/mm^2^) with five repeats of the b0 (no diffusion weighted image). The imaging parameters were 75 continuous axial slices with a slice thickness of 2 mm and no gap, field of view (FOV) = 256*256 mm^2^; TR = 9400 ms; TE = 84 ms; and matrix size = 128 × 128, resulting in 2 mm isotropic voxels.

For each subject, a high-resolution structural image was acquired by using a three-dimensional MRI sequence with a voxel size of 1 × 1 × 1 mm^3^ using an axial Fast Spoiled Gradient Recalled sequence (FSGPR) with the following parameters: repetition time (TR) = 1.900 ms; echo time (TE) = 2.26 ms; data matrix = 256 × 256; and field of view (FOV) = 256 × 256 mm^2^.

### Data Pre-Processing

An average b0 image (mB0) was calculated from the five unweighted b0 volumes, while 30 average DWIs were calculated based on the two repeats. Those steps were performed by using MATLAB (MathWorks, Natick, MA, USA). For the DWI data, pre-processing was all performed by using Leemans’s ExploreDTI (www.exploredti.com)^26^. Firstly, image quality was checked qualitatively. Subsequently, subject motion and EPI distortions were corrected with B-matrix rotation and signal intensity was modulated^26–30^. Lastly, brain extraction, diffusion tensor and FA calculation were completed^31^. The hFA image was estimated by using MATLAB based on the introduction from Goodlett, *et al*.^11^ (see Supplementary Materials).

For the T1 weighted image, brain extraction was performed with the FMRIB Software Library (FSL) v5.0 software (http://fsl.fmrib.ox.ac.uk/fsl)^31^.

### Tractography atlas-based analysis (TABS) method

**Figure 1:**
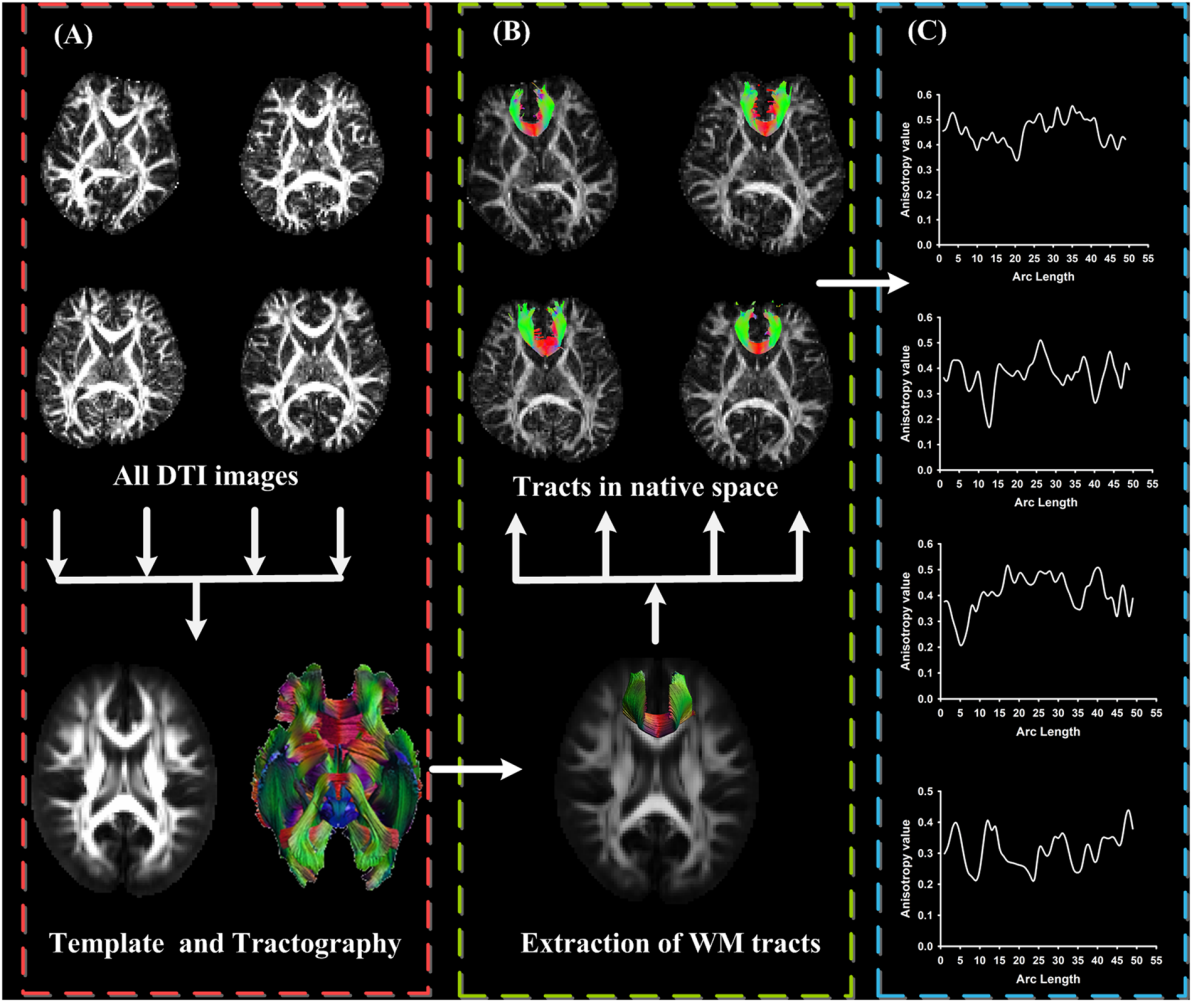
The schematic overview of the tractography atlas-based analysis. Individual tensor information integration and tractography atlas construction is shown in (**A**). Predefined fiber tract property parameterization and inverse transformation are shown in (**B**). Diffusion measure collection in native space is displayed in (**C**).

TABS is a method for group comparison of DTI combining spatial normalization of tensor images in the individual’s native space with a voxel-wise statistical framework for tract-oriented statistics^12^. By using this method, the location of the between-group differences was determined by the hypothesis test and could be investigated along a fiber pathway^12,32^. Three main steps of TABS are: individual tensor information integration and tractography atlas construction (Fig. 1A), predefined fiber tract property parameterization and inverse transformation (Fig. 1B), and diffusion measure collection in native space (Fig. 1C). Specifically:

1. Individual tensor information integration and tractography atlas construction (Fig. 1A): In order to get a common anatomical position, a number of DTI images in individual native space were transformed to a population space to get a DTI template. The multi-component DT images of each subject from individual native space were transformed with deformation fields to the population space with tensor reorientation. Then, the DT template was obtained by averaging normalized DT images. Fiber tracking was performed in this DT template using a deterministic streamline fiber tracking approach with the minimal threshold FA of 0.2 and a maximal threshold angle of 30°, which was based on the ExploreDTI software package^33,34^.
2. Predefined fiber tract property parameterization and inverse transformation (Fig. 1B): Fiber clusters in the regions of interest (ROI) were first chosen from the whole-brain tractography maps. Tract masks were created in the global-brain tractography atlas using manually defined inclusion, AND, OR, and exclusion, NOT. Then, selection of ROIs was performed manually in DT template space based on expert neuroanatomical knowledge of known pathways derived from classical anatomical descriptions. Arc length parameterization and optimal point match method were applied to define parameterized fiber clusters with a common coordinate system in population space^12^. For each subject, to get the voxel-wise coordinate matched with ROI fibers in population space, parameterized ROI tracts were transformed back to native space with inverse deformation fields.
3. Diffusion measure collection in native space (Fig. 1C): After the correspondence between individual native space and population space had been calculated, the diffusivity metrics of each voxel along the fiber pathway of each subject were extracted.

For the pipeline of the tractography atlas-based analysis, accuracy of the deformation field calculation not merely affected the accuracy of the diffusion tensor template construction but also affected the accuracy of the inverse deformation fields which were used to confirm the white matter’s relevant position in native space. Hence, the choice of feature image will influence the results of the TABS method. In our current study, we applied different feature images to research the influence on the statistical analysis in the pipeline of the TABS method.

### The deformation field calculation

To provide spatial normalization for the analysis of diffusion values at corresponding locations in TABS, deformation fields between native space and population space should be calculated first. Van *et al*.^16^ introduced a population-based registration strategy where all subject data were iteratively aligned to each other to obtain mean deformation fields of each subject image to all other images^16^. This strategy can obtain optimal spatial alignment results and has a higher accuracy and precision for transformation than the subject-based template method^16^. In the current study, we followed a similar registration strategy in Van *et al*.^16^ study. The T1 weighted image was used as an example to expound on the procedure, and the framework is elucidated in online Supplementary Fig. S1.

1. The mB0 image of each subject was first linearly aligned to the T1 weighted image to match it spatially by using FMRIB’s Linear Image Registration Tool (FLIRT)^14,35^. Affine transformation measured with the FLIRT command was obtained and named ${w}_{1}\cdots {w}_{N}$. N is the number of subjects. Subsequently, the T1 weighted images of each subject were transformed to the MNI152-T1-2mm template by using FMRIB’s FLIRT and Non-Linear Registration Tools (FNIRT)^36^. Spline coefficients ${\varphi }_{1}\cdots {\varphi }_{N}$ were obtained between T1 images and the MNI152-T1-2mm template (Fig. S1A).
2. T1 images in MNI space were iteratively non-linearly aligned to every other one to obtain the deformation fields of each subject image to all other images. The mean transformation to all other images was defined as ${\phi }_{1}\cdots {\phi }_{N}$ (Fig. S1B).
3. Consecutive application of transformations ${w}_{i}o{\varphi }_{i}o{\phi }_{i}$ (i = 1…N) was constructed based on the convertwarp command, which is an FSL tool for combining multiple transforms into one. The composite transformation ${w}_{i}o{\varphi }_{i}o{\phi }_{i}$ was defined as the final deformation field in the following analysis (Fig. S1C).

For the other two feature images (FA image and hFA image), the deformation field was calculated following similar steps except for lack of linear alignment with the mB0 image. We used the ICBM_Mori_DTI_2mm_FA template for initializing warping of the FA image. The framework is elucidated in online Supplementary Fig. S2.

### Evaluation of Inter-subject spatial normalization in simulated data sets

In order to assess accuracy and precision of tensor matching between standardized subjects, a series of simulated data sets was produced by using ground truth methodology previously presented by Van *et al*.^16^. Specifically, a random single subject DTI data set was selected as the ground truth image (GT). Then, 10 sinusoidal deformation fields were defined with different frequencies, amplitude, and direction. Another 10 sinusoidal deformation fields were the inverse of the 10 deformation fields defined before and total vector sum over all of the deformation fields equal to 0 for each voxel^37,38^. Twenty simulated data sets were deformed from the ground truth image with predefined deformation fields. Then, 20 simulated images were normalized by using different feature images. Normalized images were named T1_i,normalized_, FA_i,normalized_, and hFA_i,normalized_ respectively (i = 1…20). Normalized images more closely resembled the ground truth image indicating that they had more consistency in space alignment^16^.

Two similarity metrics were calculated to evaluate the difference in tensor matching between normalized images and the ground truth image respectively. The definitions are briefly described as follows.

*Difference in FA* is a value reflecting the similarity of 2 FA images that is defined as the accuracy and precision in the FA difference and standard deviation between the normalized image and ground truth image^11^:

1 $${\rm{FA}}\,{\rm{accuracy}}=|\frac{1}{{\rm{N}}}\sum _{{\rm{i}}=1}^{{\rm{N}}}{{\rm{FA}}}^{{{\rm{IMAGE}}}_{{\rm{i}},\text{normalized}}}-{{\rm{FA}}}^{{\rm{GT}}}|$$

2 $${\rm{FA}}\,{\rm{precision}}=|\frac{1}{{\rm{N}}}\sum _{{\rm{i}}=1}^{{\rm{N}}}{({{\rm{FA}}}^{{{\rm{IMAGE}}}_{{\rm{i}},\text{normalized}}}-{{\rm{FA}}}^{{\rm{GT}}})}^{2}|$$

where N is the number of subjects, and IMAGE_i,normalized_ is the normalized image which performed well with the T1 image, FA image, or hFA image. FA ^IMAGE^_i,normalized_ and FA^GT^ are the FA values that were derived from the normalized subject image and ground truth image in each voxel.

*The overlap of eigenvalue-eigenvector pairs (OVL)* represents the rate of orientational information preservation during image normalization, which is calculated by using^39^:

3 $${\rm{OVL}}=\frac{{\sum }_{j=1}^{3}({\lambda }_{{\rm{j}}}{\lambda }_{{\rm{j}}}^{\ast }\cdot {({\varepsilon }_{{\rm{j}}}\cdot {\varepsilon }_{{\rm{j}}}^{\ast })}^{2})}{{\sum }_{{\rm{j}}=1}^{3}{\lambda }_{{\rm{j}}}{\lambda }_{{\rm{j}}}^{\ast }}$$

4 $${\rm{OVL}}\,{\rm{accuracy}}={{\rm{OVL}}}^{{\rm{mean}}({{\rm{IMAGE}}}_{{\rm{i}},\text{normalized}}),{\rm{GT}}}$$

5 $${\rm{OVL}}\,{\rm{precision}}=\frac{1}{{\rm{N}}}\sum _{{\rm{i}}=1}^{{\rm{N}}}{{\rm{OVL}}}^{{{\rm{IMAGE}}}_{{\rm{i}},\text{normalized}},\text{GT}}$$

where ${\varepsilon }_{{\rm{j}}}$, ${\lambda }_{{\rm{j}}}$, and ${\varepsilon }_{{\rm{j}}}^{\ast }$, ${\lambda }_{{\rm{j}}}^{\ast }$ are the eigenvalue-eigenvector pairs that were derived from 2 DT images^39^. N is the number of subjects. mean (IMAGE_i,normalized_) indicates the average of all normalized images.

To replicate and validate significant findings, we randomly selected another subject as a replication data set and repeated the above analysis.

### Evaluation of Inter-subject spatial normalization in actually measured data sets

In order to corroborate the results in simulated data sets, T1_normalized_, FA_normalized_, and hFA_normalized_ were performed on 66 healthy controls to compare the difference of inter-subject spatial normalization with different feature images. A number of similarity metrics was also calculated including: difference in overlap of eigenvalue–eigenvector pairs (OVL)^39^, Euclidean distance of tensors (DTED)^40^, Euclidean distance of deviatoric tensors (DVED)^40^, angle of primary eigenvectors (AI)^15^, coherence of primary eigenvectors (COH)^41^, and cross-correlation of FA (corrFA)^15^. The definitions of these similarity metrics are described in the Supplementary Materials section.

In order to localize the sensitivity and specificity of inter-subject spatial normalization which were performed with different feature images, a series of regions of interest (ROIs) was selected as follows: anterior limb of the internal capsule (ALIC), cingulum (CG), external capsule (EC), fornix, genu of the corpus callosum (GCC), posterior limb of the internal capsule (PLIC), splenium of the corpus callosum (SCC), corticospinal tract (CST), superior longitudinal fascicle (SLF) and inferior fronto-occipital fasiculus (IFOF). A comparison of the DTED, DVED, OVL, COH, AI and corrFA was performed in selected ROIs.

### Visualization of fiber tracking

In order to access the accuracy of fiber orientation information in an intuitive way, fiber tracking visualization was performed on three diffusion tensor templates which were constructed by simulated data sets and actually measured the data sets respectively. Diffusion tensor template was constructed by averaging these normalized diffusion tensors. Three final templates were named T1_template_, FA_template_, and hFA_template_. Seed points were defined as the selected ROIs above. Deterministic streamline fiber tracking was initiated in each voxel with the minimal threshold FA of 0.2 and a maximal threshold angle of 30°^34,42^.

### Statistical analysis

To test the effect of different feature images for spatial alignment between each subject, ANOVA was performed for the evaluation value of inter-subject spatial normalization in simulated data sets and actually measured data sets. The threshold for statistical significance was *p* < 0.001(corrected by Bonferroni correction). Then, a Wilcoxon matched pairs signed rank test was used to investigate the difference of the underlying tensor information of spatial alignment between each subject.

Two-sample *t*-test was employed to detect the group difference of the anisotropy value along fibers between the two groups. Threshold-free cluster enhancement was used to obtain continuous space differences, and family-wise error (FWE) rate was applied for correcting the multiple comparisons along the fibers^24,43^.

### Sensitivity and specificity along a fiber tract

Gender difference in the corpus callosum has been repeatedly reported in previous research^44–46^. To calculate the sensitivity of the TABS method with different feature images, the tract-averaged estimate method was first used to confirm former achievements on gender difference^47^. Subsequently, a tractography atlas-based analysis was used to detect the locations which have significant differences in FA values.

In order to demonstrate the specificity of the gender difference in white matter of the corpus callosum, gender classification can be calculated with the ROC curve which is a widely used tool for comprehensive description of diagnostic accuracy^48,49^. The area under the curve (AUC) indicates the diagnostic accuracy of a classification feature between the differences of the 2 groups^49^. In the current study, local FA values had a significant difference between the male and female group in the corpus callosum fibers that were extracted as a classification feature.

## Results

### Inter-subject spatial normalization in simulated data sets

**Figure 2:**
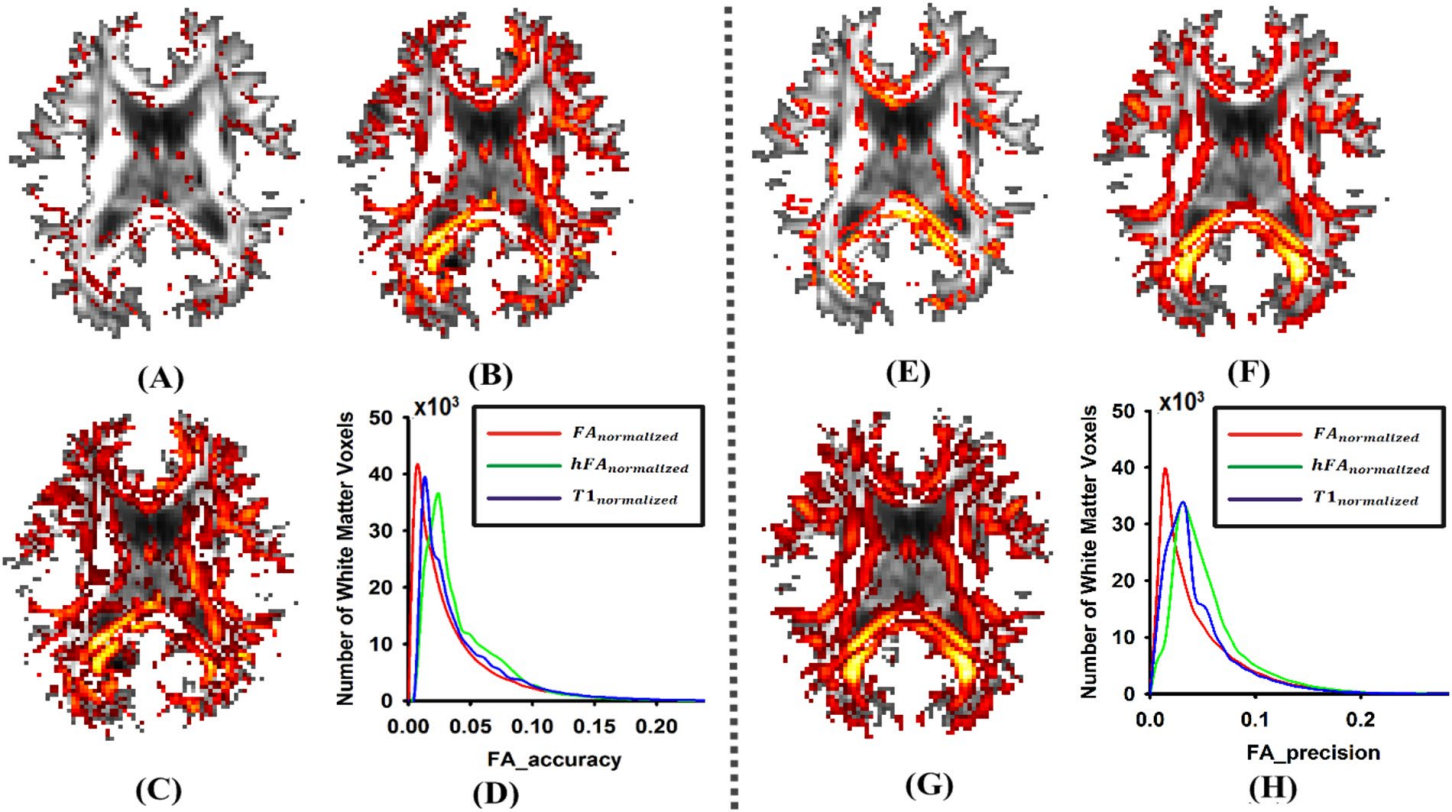
The results of the absolute value of the FA difference. (**A**,**B** and **C**) are the *accuracy of the FA difference* between the ground truth image and the normalized image which were obtained by using the FA image, T1 weighed image and hFA image as the feature image respectively. (**E**,**F** and **G**) are the *precision values of the FA difference* between the ground truth image and the normalized image, which was obtained by using the FA image, T1 weighed image and hFA image as the feature image respectively. In (**D**) and (**H**), histograms of the relative number of brain voxels corresponding to FA’s different accuracies and precisions are displayed.

To investigate which feature image was more accurate with respect to the underlying tensor information, the FA difference between normalized images and ground truth image was calculated for each voxel. As can be seen from Fig. 2, the accuracy of the FA difference was lowest in FA_normalized_ (Fig. 2A), intermediate in T1_normalized_ (Fig. 2B), and highest in hFA_normalized_ (Fig. 2C); the precision of FA differences also revealed ordered differences such that FA_normalized_ < T1_normalized_ < hFA_normalized_ (Fig. 2E–G). Histograms further confirmed these results, which were statistically significant (Fig. 2D,H, one-way ANOVA, *p* < 10^−10^).

**Figure 3:**
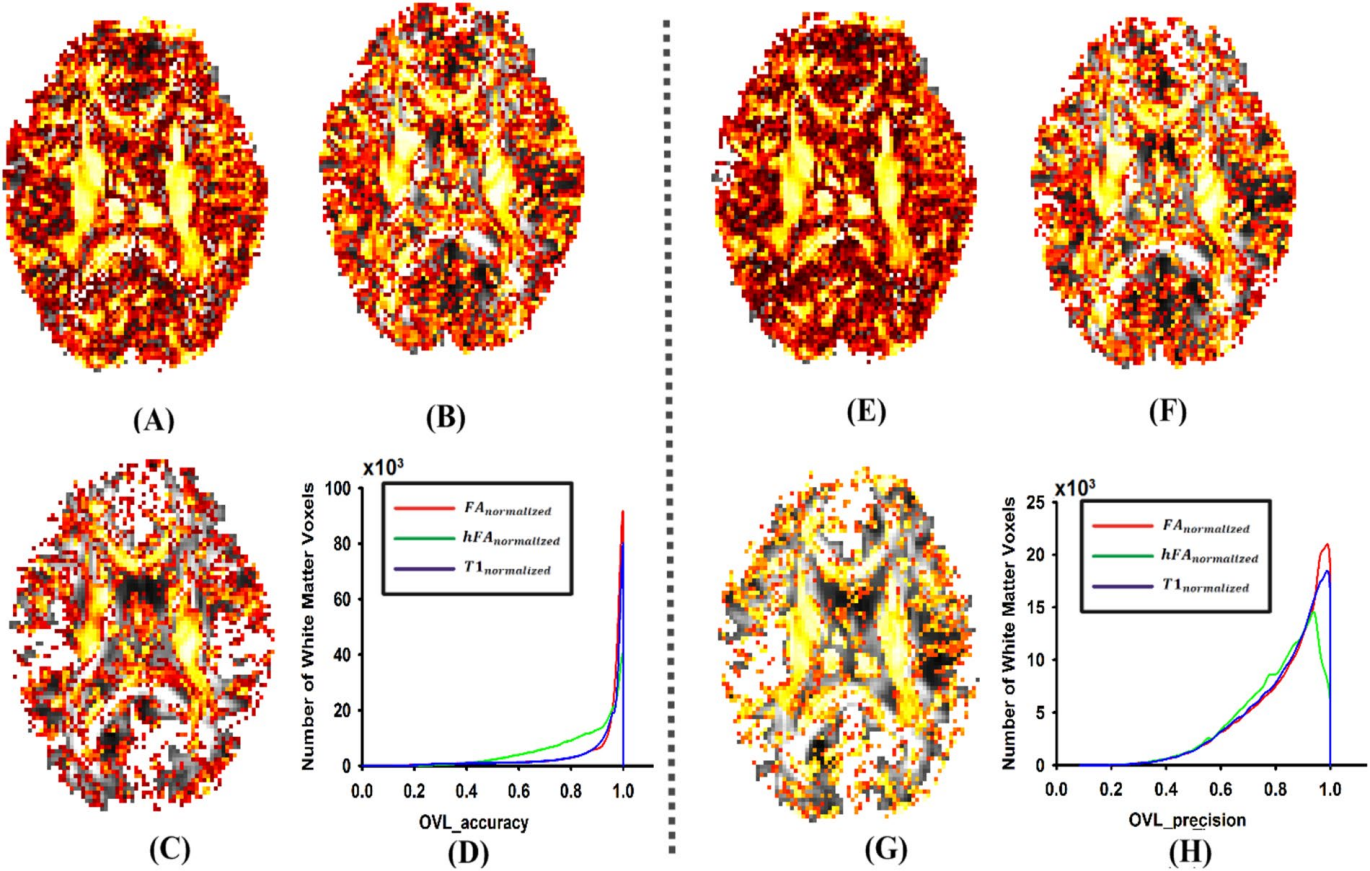
The results of the overlap of eigenvalue–eigenvector pairs. (**A**,**B** and **C**) are the *accuracies of the OVL difference* between the ground truth image and normalized image which were obtained by using the FA image, T1 weighed image and hFA image as the feature image respectively. (**E**,**F** and **G**) are the *precisions of the OVL difference* between the ground truth image and normalized image, which were obtained by using the FA image, T1 weighed image and hFA image as the feature image respectively. In (**D**) and (**H**), histograms of the relative number of brain voxels corresponding to different values of the OVL accuracy and precision are displayed.

In addition, to investigate which feature image was more sensitive with respect to orientation information of the tensor image, OVL difference between the 3 templates and ground truth image was also calculated for each voxel. As can be seen from Fig. 3, the accuracy of OVL was highest in FA_normalized_ (Fig. 3A), intermediate in T1_normalized_ (Fig. 3B), and lowest in hFA_normalized_ (Fig. 3C); the precision of OVL also revealed ordered differences such that FA_normalized_ > T1_normalized_ > hFA_normalized_ (Fig. 3E–G). Histograms further confirmed these results, which were statistically significant (Fig. 3D,H, one-way ANOVA, *p* < 10^−10^).

An independent replication data set was included to replicate and validate significant findings. In our study, calculation of the FA difference and OVL for an independent replication data set had similar results.

### Inter-subject spatial normalization in actually measured data sets

**Figure 4:**
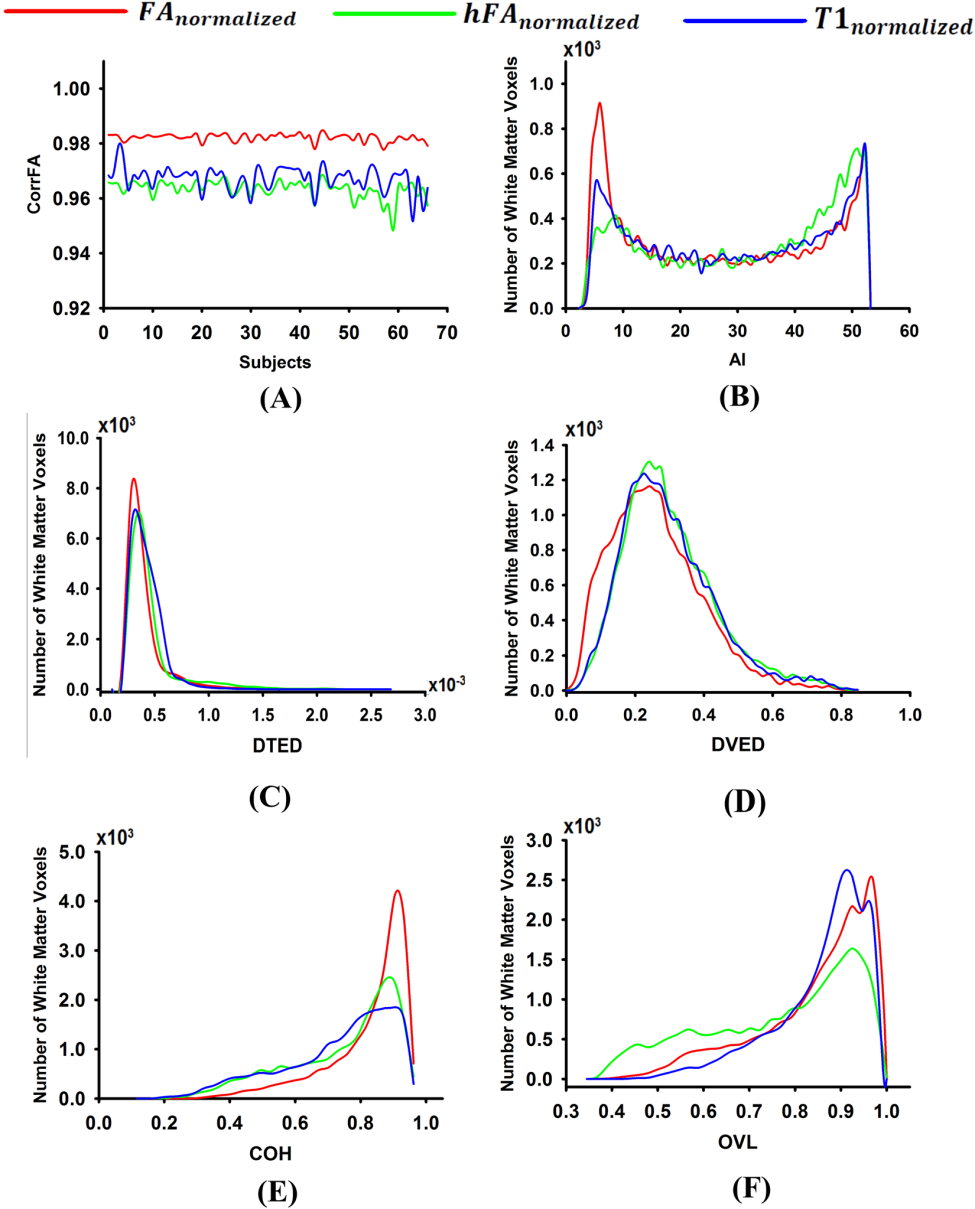
Histograms of the relative number of brain voxels corresponding to different values of the cross-correlation of FA (corrFA) (**A**), the angle of primary eigenvectors (AI) (**B**), the Euclidean distance of tensors (DTED) (**C**), the Euclidean distance of the deviatoric tensors (DVED) (**D**), the coherence of primary eigenvectors (COH) (**E**), and overlap of eigenvalue–eigenvector pairs (OVL) (**F**) for FA_normalized_ (red curve), hFA_normalized_ (green curve), and T1_normalized_ (blue curve).

As can be seen from Fig. 4(A), a greater mean corrFA value was present in FA_normalized_ than in T1_normalized_ and a lesser mean corrFA value was present in hFA_normalized_. In the pairwise comparison of the 3 templates, FA_normalized_ demonstrated the largest percentage of voxels with low AI (Fig. 4B), low DTED (Fig. 4C), low DVED (Fig. 4D), high COH (Fig. 4E), and high OVL (Fig. 4F) values, and a minimum percentage of voxels with high AI (Fig. 4B), high DTED (Fig. 4C), high DVED (Fig. 4D), low COH (Fig. 4E), and low OVL (Fig. 4F) values. Meanwhile, the histogram of T1_normalized_ and hFA_normalized_ revealed the transition T1_normalized_ > hFA_normalized_ (Fig. 4A–F) (one-way ANOVA, *p* < 10^−10^).

As can be seen from Tables 1–6, mean corrFA (Table 1), OVL (Table 2) and COH (Table 3) in white matter ROIs for the 3 templates revealed the transition FA_normalized_ > T1_normalized_ > hFA_normalized_ (*p* < 10^−10^). On the contrary, mean DTED (Table 4), DVED (Table 5) and AI (Table 6) in white matter ROIs for the 3 templates revealed the transition FA_normalized_ < T1_normalized_ < hFA_normalized_ (one-way ANOVA, *p* < 10^−10^). These were more similar to the results of the evaluation in the actually measured data sets.

**Table 1:** Average corrFA of tensors over all pairs of datasets used in T1_normalized_, FA_normalized_, and hFA_normalized_.

Brain Region	FA_normalized_		T1_normalized_		hFA_normalized_		*F*	*P*	*P1*	*P2*	*P3*
	mean	STD	mean	STD	mean	STD					
ALIC	0.985	0.002	0.982	0.008	0.974	0.002	143.681	<10^−10^	<10^−10^	<10^−10^	<10^−10^
CG	0.961	0.007	0.919	0.022	0.899	0.004	287.254	<10^−10^	<10^−10^	<10^−10^	<10^−10^
EC	0.982	0.002	0.976	0.009	0.965	0.023	99.574	<10^−10^	<10^−10^	<10^−10^	<10^−10^
fornix	0.963	0.015	0.951	0.015	0.940	0.019	62.199	<10^−10^	<10^−10^	<10^−10^	<10^−10^
GCC	0.965	0.002	0.963	0.010	0.963	0.017	91.733	<10^−10^	<10^−10^	<10^−10^	<10^−10^
PLIC	0.995	0.001	0.994	0.005	0.988	0.001	101.240	<10^−10^	<10^−10^	<10^−10^	<10^−10^
SCC	0.979	0.003	0.972	0.018	0.952	0.015	85.627	<10^−10^	<10^−10^	<10^−10^	<10^−10^
CST	0.988	0.001	0.981	0.004	0.975	0.002	48.548	<10^−10^	<10^−10^	<10^−10^	<10^−10^
SLF	0.963	0.005	0.961	0.008	0.942	0.007	215.958	<10^−10^	<10^−10^	<10^−10^	<10^−10^
IFOF	0.985	0.004	0.977	0.007	0.974	0.006	40.357	<10^−10^	<10^−10^	0.021	<10^−10^

ALIC: anterior limb of the internal capsule, CG: cingulum, EC: external capsule, fornix, GCC: genu of the corpus callosum, PLIC: posterior limb of the internal capsule, SCC: splenium of the corpus callosum, CST: corticospinal tract, SLF: superior longitudinal fascicle, IFOF: inferior fronto-occipital fasiculus. corrFA: cross-correlation of FA. ANOVA was used to compare the difference between the three diffusion tensor templates. *P1*: Statistically significant differences between FA_normalized_ and T1_normalized_ (after correction for multiple comparisons with the Bonferroni approach). *P2*: Statistically significant differences between T1_normalized_ and hFA_normalized_ (after correction for multiple comparisons with the Bonferroni approach). *P3*: Statistically significant differences between FA_normalized_ and hFA_normalized_ (after correction for multiple comparisons with the Bonferroni approach).

**Table 2:** Average OVL of tensors over all pairs of datasets used in T1_normalized_, FA_normalized_, and hFA_normalized_.

Brain Region	FA_normalized_		T1_normalized_		hFA_normalized_		*F*	*P*	*P1*	*P2*	*P3*
	mean	STD	mean	STD	mean	STD					
ALIC	0.955	0.157	0.917	0.063	0.897	0.109	198.688	<10^−10^	<10^−10^	<10^−10^	<10^−10^
CG	0.782	0.054	0.688	0.135	0.604	0.101	400.174	<10^−10^	<10^−10^	<10^−10^	<10^−10^
EC	0.850	0.052	0.849	0.090	0.830	0.147	85.812	<10^−10^	<10^−10^	<10^−10^	<10^−10^
fornix	0.875	0.097	0.814	0.070	0.800	0.108	88.842	<10^−10^	<10^−10^	<10^−10^	<10^−10^
GCC	0.820	0.104	0.796	0.150	0.774	0.145	151.287	<10^−10^	<10^−10^	<10^−10^	<10^−10^
PLIC	0.945	0.105	0.925	0.056	0.899	0.063	75.634	<10^−10^	<10^−10^	<10^−10^	<10^−10^
SCC	0.847	0.120	0.842	0.119	0.839	0.124	33.732	<10^−10^	0.061	<10^−10^	<10^−10^
CST	0.852	0.056	0.839	0.099	0.798	0.175	173.483	<10^−10^	<10^−10^	<10^−10^	<10^−10^
SLF	0.710	0.162	0.703	0.132	0.605	0.166	167.277	<10^−10^	0.030	<10^−10^	<10^−10^
IFOF	0.854	0.045	0.851	0.096	0.812	0.098	5.554	0.015	0.059	<10^−10^	<10^−10^

ALIC: anterior limb of the internal capsule, CG: cingulum, EC: external capsule, fornix, GCC: genu of the corpus callosum, PLIC: posterior limb of the internal capsule, SCC: splenium of the corpus callosum, CST: corticospinal tract, SLF: superior longitudinal fascicle, IFOF: inferior fronto-occipital fasiculus. OVL: overlap of eigenvalue–eigenvector pairs. ANOVA was used to compare the difference between the three diffusion tensor templates. *P1*: Statistically significant differences between FA_normalized_ and T1_normalized_ (after correction for multiple comparisons with the Bonferroni approach). *P2*: Statistically significant differences between T1_normalized_ and hFA_normalized_ (after correction for multiple comparisons with the Bonferroni approach). *P3*: Statistically significant differences between FA_normalized_ and hFA_normalized_ (after correction for multiple comparisons with the Bonferroni approach).

**Table 3:** Average COH of tensors over all pairs of datasets used in T1_normalized_, FA_normalized_, and hFA_normalized_.

Brain Region	FA_normalized_		T1_normalized_		hFA_normalized_		*F*	*P*	*P1*	*P2*	*P3*
	mean	STD	mean	STD	mean	STD					
**ALIC**	0.890	0.047	0.879	0.049	0.834	0.105	180.431	<10^−10^	<10^−10^	<10^−10^	<10^−10^
**CG**	0.749	0.126	0.660	0.156	0.659	0.147	179.477	<10^−10^	<10^−10^	<10^−10^	<10^−10^
**EC**	0.853	0.092	0.833	0.085	0.810	0.099	105.047	<10^−10^	<10^−10^	<10^−10^	<10^−10^
**fornix**	0.864	0.052	0.847	0.081	0.765	0.196	99.418	<10^−10^	0.087	<10^−10^	<10^−10^
**GCC**	0.848	0.097	0.782	0.137	0.722	0.162	216.864	<10^−10^	<10^−10^	<10^−10^	<10^−10^
**PLIC**	0.912	0.030	0.890	0.049	0.876	0.174	57.977	<10^−10^	<10^−10^	<10^−10^	<10^−10^
**SCC**	0.844	0.126	0.814	0.109	0.800	0.193	332.911	<10^−10^	<10^−10^	<10^−10^	<10^−10^
**CST**	0.823	0.107	0.798	0.101	0.732	0.103	166.223	<10^−10^	<10^−10^	<10^−10^	<10^−10^
**SLF**	0.701	0.150	0.671	0.140	0.595	0.167	155.456	<10^−10^	0.007	<10^−10^	<10^−10^
**IFOF**	0.839	0.116	0.833	0.081	0.799	0.155	6.764	0.031	0.603	0.0005	<10^−10^

ALIC: anterior limb of the internal capsule, CG: cingulum, EC: external capsule, fornix, GCC: genu of the corpus callosum, PLIC: posterior limb of the internal capsule, SCC: splenium of the corpus callosum, CST: corticospinal tract, SLF: superior longitudinal fascicle, IFOF: inferior fronto-occipital fasiculus. COH: coherence of primary eigenvectors. ANOVA was used to compare the difference between the three diffusion tensor templates. *P1*: Statistically significant differences between FA_normalized_ and T1_normalized_ (after correction for multiple comparisons with the Bonferroni approach). *P2*: Statistically significant differences between T1_normalized_ and hFA_normalized_ (after correction for multiple comparisons with the Bonferroni approach). *P3*: Statistically significant differences between FA_normalized_ and hFA_normalized_ (after correction for multiple comparisons with the Bonferroni approach).

**Table 4:** Average DTED of tensors over all pairs of datasets used in T1_normalized_, FA_normalized_, and hFA_normalized_.

Brain Region	F_Anormalized_ × 10^−4^		T1_normalized_ × 10^−4^		hFA_normalized_ × 10^−4^		*F*	*P*	*P1*	*P2*	*P3*
	mean	STD	mean	STD	mean	STD					
ALIC	2.147	0.418	2.532	0.401	2.614	0.413	59.417	<10^−10^	<10^−10^	<10^−10^	<10^−10^
CG	2.041	0.359	3.338	0.437	3.813	0.570	98.674	<10^−10^	<10^−10^	<10^−10^	<10^−10^
EC	1.998	0.334	2.446	0.416	2.562	0.820	385.748	<10^−10^	<10^−10^	0.013	<10^−10^
fornix	7.355	0.975	7.453	1.450	8.042	0.292	43.742	<10^−10^	0.0003	0.004	<10^−10^
GCC	4.419	2.447	4.432	1.352	4.504	2.468	2.174	0.227	—	—	—
PLIC	2.365	0.287	2.719	0.280	2.922	0.597	162.217	<10^−10^	<10^−10^	<10^−10^	<10^−10^10^−10^
SCC	3.376	1.811	4.539	0.954	4.983	3.075	278.081	<10^−10^	0.187	<10^−10^	<10^−10^
CST	2.477	0.735	2.830	0.434	3.101	0.743	546.853	<10^−10^	<10^−10^	<10^−10^	<10^−10^
SLF	3.494	0.797	3.567	0.645	3.813	0.519	54.659	<10^−10^	<10^−10^	<10^−10^	<10^−10^
IFOF	2.057	0.642	2.627	0.322	2.909	0.742	100.459	<10^−10^	<10^−10^	<10^−10^	<10^−10^

ALIC: anterior limb of the internal capsule, CG: cingulum, EC: external capsule, fornix, GCC: genu of the corpus callosum, PLIC: posterior limb of the internal capsule, SCC: splenium of the corpus callosum, CST: corticospinal tract, SLF: superior longitudinal fascicle, IFOF: inferior fronto-occipital fasiculus. DTED: Euclidean distance of tensors. ANOVA was used to compare the difference between the three diffusion tensor templates. *P1*: Statistically significant differences between FA_normalized_ and T1_normalized_ (after correction for multiple comparisons with the Bonferroni approach). *P2*: Statistically significant differences between T1_normalized_ and hFA_normalized_ (after correction for multiple comparisons with the Bonferroni approach). *P3*: Statistically significant differences between FA_normalized_ and hFA_normalized_ (after correction for multiple comparisons with the Bonferroni approach).

**Table 5:** Average DVED of tensors over all pairs of datasets used in T1_normalized_, hFA_normalized_, and hFA_normalized_.

Brain Region	FA_normalized_ × 10^−4^		T1_normalized_ × 10^−4^		hFA_normalized_ × 10^−4^		*F*	*P*	*P1*	*P2*	*P3*
	mean	STD	mean	STD	mean	STD					
ALIC	2.233	0.475	2.248	0.609	2.579	0.288	55.768	<10^−10^	<10^−10^	<10^−10^	<10^−10^
CG	2.485	0.282	2.903	0.466	3.001	0.357	187.456	<10^−10^	<10^−10^	0.457	<10^−10^
EC	2.080	0.378	2.169	0.420	2.476	0.366	162.751	<10^−10^	<10^−10^	0.003	<10^−10^
fornix	3.002	0.468	3.517	0.648	3.898	0.301	220.635	<10^−10^	<10^−10^	<10^−10^	<10^−10^
GCC	2.593	0.739	2.983	0.652	3.722	0.511	512.471	<10^−10^	<10^−10^	<10^−10^	<10^−10^
PLIC	2.506	0.317	2.545	0.645	2.922	0.238	102.876	<10^−10^	<10^−10^	<10^−10^	<10^−10^
SCC	2.999	0.384	3.173	0.714	3.992	0.693	664.578	<10^−10^	0.0002	<10^−10^	<10^−10^
CST	2.579	0.379	2.676	0.735	3.247	0.835	323.854	<10^−10^	<10^−10^	<10^−10^	<10^−10^
SLF	2.874	0.274	3.066	0.775	3.454	0.398	164.599	<10^−10^	<10^−10^	<10^−10^	<10^−10^
IFOF	2.001	0.462	2.121	0.239	2.777	0.778	311.526	<10^−10^	<10^−10^	<10^−10^	<10^−10^

ALIC: anterior limb of the internal capsule, CG: cingulum, EC: external capsule, fornix, GCC: genu of the corpus callosum, PLIC: posterior limb of the internal capsule, SCC: splenium of the corpus callosum, CST: corticospinal tract, SLF: superior longitudinal fascicle, IFOF: inferior fronto-occipital fasiculus. DVED: Euclidean distance of the deviatoric tensors. ANOVA was used to compare the difference between the three diffusion tensor templates. *P1*: Statistically significant differences between FA_normalized_ and T1_normalized_ (after correction for multiple comparisons with the Bonferroni approach). *P2*: Statistically significant differences between T1_normalized_ and hFA_normalized_ (after correction for multiple comparisons with the Bonferroni approach). *P3*: Statistically significant differences between FA_normalized_ and hFA_normalized_ (after correction for multiple comparisons with the Bonferroni approach).

**Table 6:** Average AI of tensors over all pairs of datasets used in T1_normalized_, FA_normalized_, and hFA_normalized_.

Brain Region	FA_normalized_		T1_normalized_		hFA_normalized_		*F*	*P*	*P1*	*P2*	*P3*
	mean	STD	mean	STD	mean	STD					
ALIC	0.687	0.355	0.733	0.459	0.774	0.264	34.240	<10^−10^	0.042	<10^−10^	<10^−10^
CG	0.993	0.344	1.110	0.298	1.145	0.763	96.273	<10^−10^	<10^−10^	<10^−10^	<10^−10^
EC	1.001	0.414	1.081	0.341	1.342	0.783	100.863	<10^−10^	<10^−10^	<10^−10^	<10^−10^
fornix	0.869	0.874	0.960	0.288	1.356	0.234	176.524	<10^−10^	0.029	<10^−10^	<10^−10^
GCC	1.082	0.267	1.110	0.357	1.225	0.675	2.987	0.078	—	—	—
PLIC	1.002	0.743	1.079	0.366	1.385	0.273	20.739	<10^−10^	0.001	0.004	<10^−10^
SCC	1.040	0.566	1.056	0.325	1.198	0.637	78.724	<10^−10^	0.199	<10^−10^	<10^−10^
CST	0.996	0.757	1.180	0.301	1.302	0.845	34.572	<10^−10^	0.038	<10^−10^	<10^−10^
SLF	0.809	0.265	0.883	0.496	0.893	0.261	1.547	0.221	—	—	—
IFOF	1.044	0.684	1.118	0.331	1.188	0.632	2.889	0.095	—	—	—

ALIC: anterior limb of the internal capsule, CG: cingulum, EC: external capsule, fornix, GCC: genu of the corpus callosum, PLIC: posterior limb of the internal capsule, SCC: splenium of the corpus callosum, CST: corticospinal tract, SLF: superior longitudinal fascicle, IFOF: inferior fronto-occipital fasiculus. AI: angle of primary eigenvectors. ANOVA was used to compare the difference between the three diffusion tensor templates. *P1*: Statistically significant differences between hFA_*template*_ and T1_normalized_ (after correction for multiple comparisons with the Bonferroni approach). *P2*: Statistically significant differences between T1_normalized_ and hFA_normalized_ (after correction for multiple comparisons with the Bonferroni approach). *P3*: Statistically significant differences between FA_normalized_ and hFA_normalized_ (after correction for multiple comparisons with the Bonferroni approac.

### Visualization of fiber tracking

**Figure 5:**
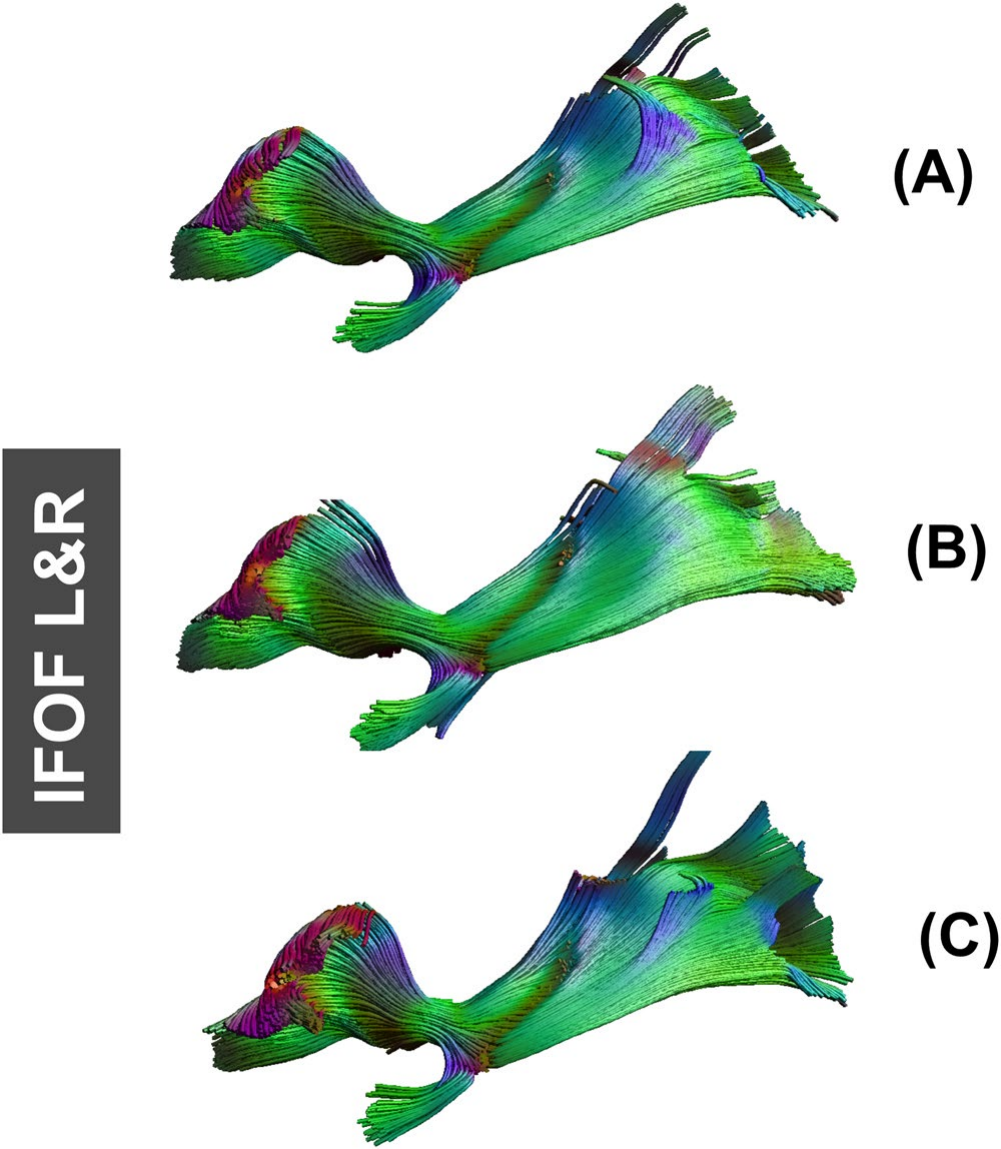
The results of fiber tracking at seed points of IFOF for FA_template_ (above), hFA_template_ (below), and T1_template_ (middle) visualized in (**A**,**B**).

**Figure 6:**
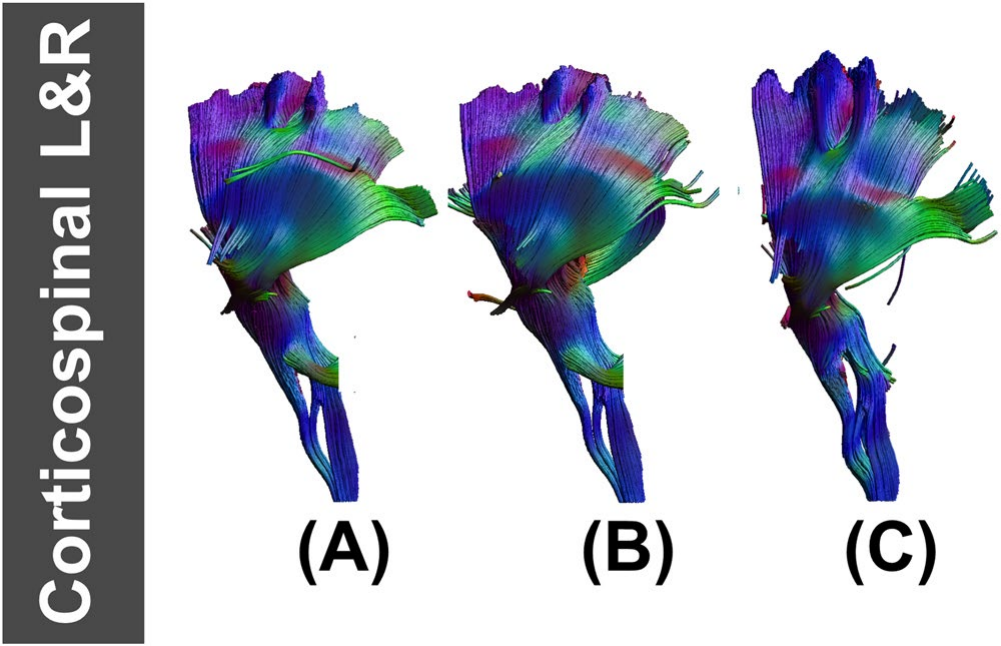
The result of fiber tracking at seed points of CST for FA_template_ (above), hFA_template_ (below), and T1_template_ (middle) visualized in (**A**,**B**).

**Figure 7:**
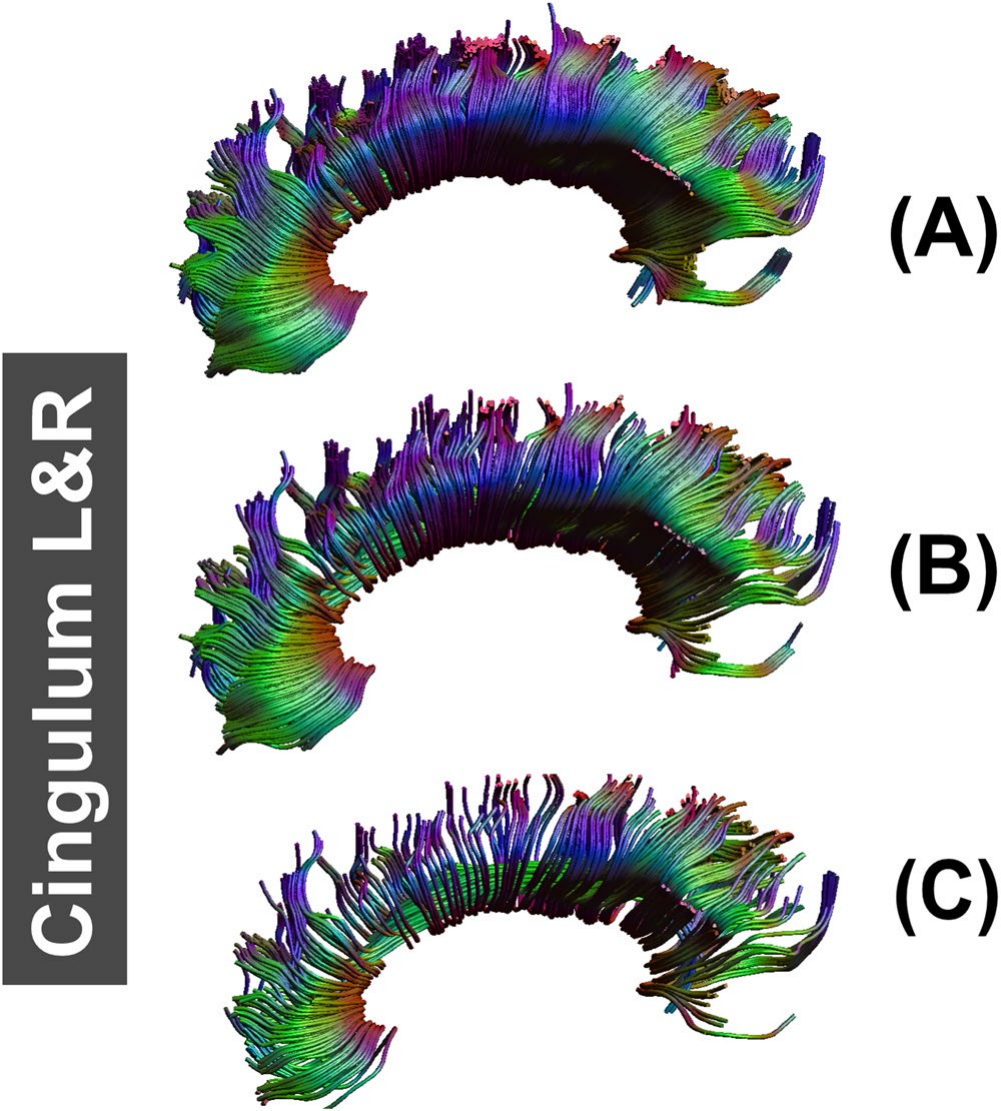
The result of fiber tracking at seed points of CG for FA_template_ (above), hFA_template_ (below), and T1_template_ (middle) which was constructed by simulated data sets and visualized in (**A**–**C**).

**Figure 8:**
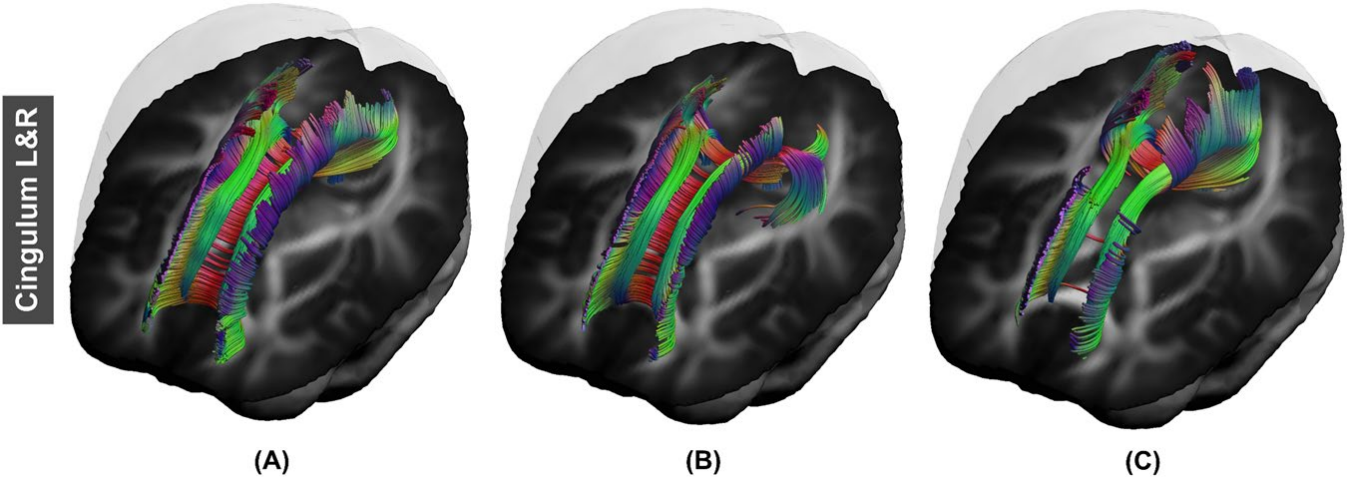
The result of fiber tracking at seed points of CG for FA_template_ (left), hFA_template_ (right), and T1_template_ (middle) which was constructed by actually measured data sets and are visualized in (**A**–**C**).

Fiber tracking results in ALIC, EC, fornix, GCC, PLIC, SCC, CST, SLF, and IFOF were similar in fiber length and sparsity. Figure 5 shows examples of fiber tracking at seed points for CST and IFOF. The fiber bundle which was reconstructed from FA_template_ (Figs 5A, 6A), T1_template_ (Figs 5B and 6B) and hFA_template_ (Figs 5C and 6C) was quite similar visually. However, for the simulated data sets, FA_template_ had the most dense white matter pathways (Fig. 7A) than T1_template_ (Fig. 7B) and hFA_template_ (Fig. 7C) in the tractography results of CG. hFA_template_ (Fig. 7C) had the most sparse white matter pathways. On the other hand, the tractography results for CG of different templates in the actually measured data sets further confirmed these results, which are displayed in Fig. 8.

### Sensitivity and specificity along a fiber tract

**Figure 9:**
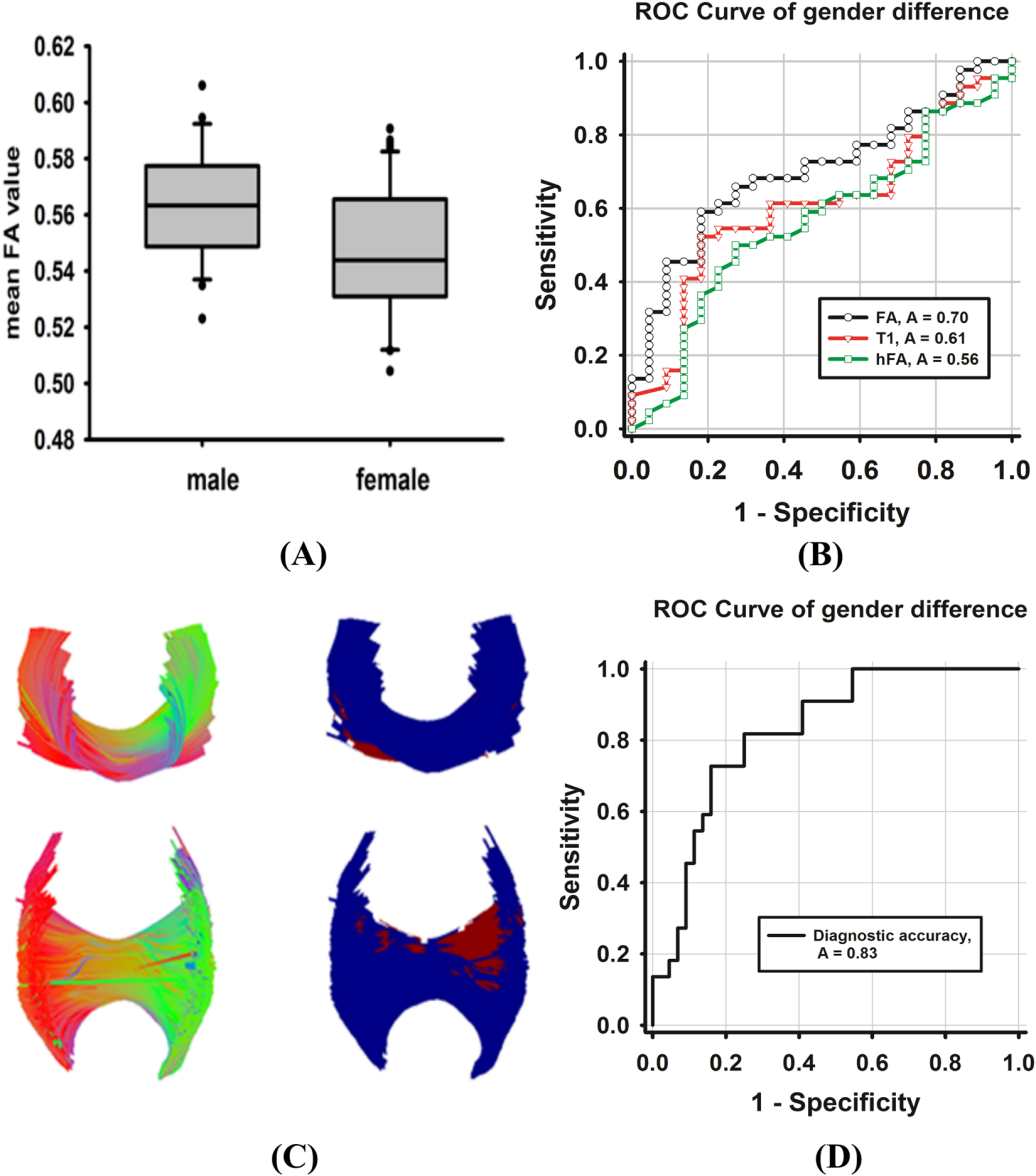
The results of gender difference and classification. In (**A**), the boxplot of the mean FA which was calculated by using the tract-averaged estimate method is displayed. ROC curve and AUC for evaluating the diagnosis of the gender difference with the tract-averaged estimate method is shown in (**B**). In (**C**), the local fiber tract which reflects the gender difference is visualized by using the along-tract group analysis. ROC curve and AUC for evaluating the diagnosis of gender difference with the along-tract group analysis is shown in (**D**).

For the between group comparison of the mean FA value of the corpus callosum fibers, there was no significant difference in the FA value between the male group and female group using the T1 image or hFA image as a feature image. While the FA image was used as a feature image, gender difference in the FA value could be found in the corpus callosum (*p* = 0.043) (Fig. 9A). Meanwhile, ROC for discriminating between the male group and female group was 0.56 for the hFA image, 0.61 for the T1 image and 0.7 for the FA image (Fig. 9B).

For the voxel-wise comparison, no significant results were found in the T1 and hFA image. Moreover, WM morphology difference in the FA value could be found in the right corpus callosum with the TABS method when using FA as a feature image (*p* < 0.05, FWE correction, Fig. 9C). Local FA values which have a significant difference in the corpus callosum fibers were extracted as a classification feature, and the ROC curve showed good diagnostic performance as shown in Fig. 9 (AUC = 0.83).

## Discussion

In this work, we tested the effect of different feature images on the sensitivity of the statistical analysis in the pipeline of the TABS method. A ground truth methodology and a series of evaluation parameters were used to investigate which feature image was more accurate with respect to the underlying tensor information for the TABS method. The results of simulated data sets showed that FA_normalized_ had the minimum difference in FA and highest consistency in orientational information than T1_normalized_ and hFA_normalized_. Simultaneously, the results of actually measured data sets revealed that FA_normalized_ also exhibited highest similarity and accuracy for scalar and vector information. For the results of the tract analysis, only when the FA image was applied to the TABS pipeline to be a feature image, the WM morphology difference in the FA value could be detected in the right corpus callosum. Our results reflected that selection of feature images would influence the quantitative statistical analysis in the pipeline of the TABS method.

Prior DTI analysis was mainly focused on scalar indices in a common coordinate system overlooking comprehensive direction information, which is a key factor in deducing brain connectivity^15,50,51^. The TABS method, as an optimized white matter analysis which creates a voxel-wise statistical framework for detecting and understanding white matter differences along a fiber tract, is advantageous in terms of detecting a WM morphology difference^12^. The 2 key procedures of the TABS method are diffusion tensor template construction and statistical model building^12–14,52,53^. The accuracy of the 2 steps has great influence in the quantitative analysis along the fiber tract. However, the feature image as one of the key steps which affects the accuracy of the 2 key procedures of the TABS method was often overlooked. Hence, precise comparison of the effect of different feature images for the accuracy of the statistical analysis in the pipeline of the TABS method is necessary for DTI analysis.

For the results of all of the similarity metrics, we noticed that FA_normalized_ showed the lowest DTED, lowest DVED, lowest AI, highest OVL, highest COH, and highest *corr*_*FA*_ than T1_normalized_ and hFA_normalized_. DTED and DVED were used to evaluate the space distance of the 2 tensors at each voxel^15,40^. Alexander *et al*.^40^ and Zhang *et al*.^15^ used them to assess the accuracy of tensor matching during image spatial normalization^15,40^. As the lower values were related to a shorter Euclidean distance in each voxel for the 2 tensors, our findings indicated that DTI datasets that were registered through the FA image could achieve a more similar spatial location at each voxel than the hFA image and T1 image. OVL, COH, and AI were parameters used to assess directions and angles of the diffusion tensor in each voxel, and several studies used them to evaluate accuracy of the coherence of tensor orientation^15,16^. Higher OVL and COH, and lower AI expressed more orientational information preservation of the 2 tensors during image normalization^15,16,39,41^. Our findings indicated that the transformation which was estimated from the FA image could obtain a higher coherence of direction in the diffusion tensor than the T1 image and hFA image. Meanwhile, corrFA is a parameter used to assess the correlation of each normalized image for the FA value. Higher values signify a higher mutual correlation of FA values between the FA image in individual datasets and the FA image derived from the normalized image^15^. The higher value observed in FA_normalized_ means that the FA image could better represent features of individuals in scalar terms than the T1 image and hFA image. According to the above information, the selection of the feature image can largely impact the quality of image normalization. Compared to the T1 image and hFA image, we found that the FA image had strong robustness to recognize white matter passageways and the microstructure could obtain the most accurate scalar information and most consistent vector information.

According to many studies, in order to register individual diffusion weighted images to a standard template, the FA image was a widely used feature image to obtain the transformation^6,14,15^. For example, Kohannim *et al*.^23^ registered the FA image to ICBM space with the FSL package to ensure spatial consistency to obtain an accurate statistical comparison^23^. To develop accuracy of the DTI analysis, Zhang *et al*.^15^ transformed subject images to standard space by means of an FA image^15^. In addition, Liu *et al*.^54^ also showed that the FA feature image is the best scalar feature for spatial normalization compared with all other scalar measurements^54^. Previous research results were broadly in line with our hypothesis that the FA image has ultra-sensitivity for identifying corresponding regions of white matter geometry and could obtain a more accurate statistical analysis with the TABS method when used as a feature image. On the other hand, structural information of the T1 image is homogeneous in white matter regions, so the sensitivity of identifying white matter geometry is smaller than the FA image^55^. Hence, compared with the FA image, the T1 image is inadequate to achieve sufficient WM alignment when registering to MNI space. Moreover, Peng *et al*.^14^ stated that DTI images were not appropriate for using transformations which were estimated based on T1 images due to the mismatch between image contrasts and resolution^14^. Saad *et al*.^56^ suggested that registration with DTI and T1 image could not produce satisfactory results, because of the mismatch between volumes^56^. These viewpoints are also broadly in line with our results that the T1 image could not get a more accurate space alignment than the FA image.

To demonstrate the sensitivity and specificity of white matter with a different feature image, a tract-averaged estimate method and the TABS method were used to detect the gender difference with the FA value in the corpus callosum^10,12^. Only when the FA image was used as a feature image, the tract-averaged estimate method could confirm the former achievements on gender difference. Furthermore, WM morphology difference in the right corpus callosum was detected by using the TABS method with the FA image as a feature image. In addition, the local FA extracted from the right corpus callosum could obtain good classification accuracy as a feature to formulate gender classification. Our results indicated that the FA image has ultra-sensitivity for identifying corresponding regions of white matter geometry than the T1 image and hFA image. Hence, in the pipeline of the TABS method, the FA image could obtain more accurate space alignment and more consistent vector information during diffusion tensor construction. On the other hand, a more sensitive feature image could improve cross-subject point-wise alignment during point correspondence along a fiber tract. Due to more accurate tensor matching, the FA image as a feature image could obtain a more accurate statistical model in the pipeline of the TABS method.

There are some limitations that should be noticed in our studies. The DTI scan was applied along 30 non-collinear directions with 5 acquisitions without diffusion weighting in our study. The limitation of DTI is the delineation of crossing and touching fibers due to insufficient anisotropy and angular separation^57^. In recent years, diffusion spectrum imaging (DSI) was increasingly applied and explored in neuroimaging studies with a higher angular resolution and more diffusion weighted directions than DTI^58–61^. In further research, the use of DSI could be a means of image scanning to get a more accurate tract analysis. In our results, it is particularly noteworthy that all of the comparisons between the feature images were based on the same image modality, which was comprised of 30 diffusion weighted images and 5 b0 images. The results in our study were independent of the type of image scanning. Besides that, application of the registration algorithm in our study was B-spline based registration within the FSL package^31^. A better registration algorithm can get more consistent matching of the local tensor orientation in each voxel. An improved DTI registration algorithm, Advanced Normalization Tools (ANTs), has been proven to have a slightly higher performance than the registration algorithm within the FSL package^62^. In the future, we can apply this improved DTI registration algorithm to our study to obtain a more accurate statistical analysis^62^. However, in our study, the TABS method was based on B-spline based registration within the FSL package and the only difference was the feature image. Hence, the results in our study could show the effects of the feature image in the pipeline of the TABS method independent of the use of the registration algorithm.

In summary, we confirmed that the feature image will affect the sensitivity of the statistical analysis in the pipeline of the TABS method by affecting the spatial consistency between each subject. Our results represented image normalization performed by using the FA image as the feature image that had higher coherence of direction and more accurate analysis results with the TABS method.

## Electronic supplementary material

Supplementary Information
