# Supplementary material for: The effect of feature image on sensitivity of the statistical analysis in the pipeline of a tractography atlas-based analysis
Source: Sci Rep. 2017 Oct 4;7:12669. doi: 10.1038/s41598-017-12965-5 (PMC5627283; doi:10.1038/s41598-017-12965-5)
Supplement: Supplementary file 1 — Supplementary Information [file 41598_2017_12965_MOESM1_ESM.pdf]

The effect of feature image on sensitivity of the statistical analysis in the pipeline of a tractography atlas-based analysis

Junya Mu[a], Qing Xu[a], Jie Tian[*a], Jixin Liu[*a]

[a] *School of Life Science and Technology, Xidian University, Xi'an 710071, Peoples R China*

Supplementary methods

The deformation field calculation

The deformation field calculation when the T1 weighted image was selected as a feature image is elucidated in the following steps (Fig S1).

1. An average B0 image (mB0) calculated from five unweighted B0 volumes was used to perform the registration. Then, EPI distortions between the mB0 and T1 weighted image were corrected by using ExploreDTI (www.exploredti.com)[1].

2. The corrected mB0 image of each subject was first linearly aligned to the T1 weighted image to match it spatially by using FMRIB's Linear Image Registration Tool (FLIRT)[2]. Affine transformation measured with the FNIRT command was obtained and named $w_1 \cdots w_N$. N is the number of subjects.

3. T1 weighted images of each subject were transformed to the MNI152-T1-2mm template by using FMRIB's FLIRT and Non-Linear Registration Tools (FNIRT)[2]. Spline coefficients $\varphi_1 \ldots \varphi_N$ were obtained between the T1 images and MNI152-T1-2mm template. T1 images in MNI space (T1-MNI), $S_1 \ldots S_N$ were obtained.

4. S_i (i=1…N) was non-linearly transformed to S_j (j=1…N, j≠i) and the transformation,

T_{is} (s=1…N-1), was obtained between S_i and S_j. The mean of the deformation fields of S_i to all other images is computed as:

$$\Phi_i = \frac{1}{N-1}\sum_{i=1}^{N-1} T_{is} \quad (i = 1 \dots N, s = 1 \dots \mathrm{N} - 1) \tag{1}$$

5. Keep repeating the above step until you obtain all of the means of the deformation fields of each subject image to all other images. Finally, the average deformation field of each subject $\Phi_1 \dots \Phi_N$ was obtained.

6. The consecutive application of the transformations $w_i \circ \varphi_i \circ \phi_i$ (i=1…N) was constructed based on the converwarp command which is an FSL tool for combining multiple transforms into one. The composite transformation $w_i \circ \varphi_i \circ \phi_i$ was defined as the final deformation field in the following analysis.

The deformation field calculation when the FA/hFA image was selected as a feature image is elucidated in the following steps (Fig S2).

1. EPI distortions between the FA/hFA image and T1 weighted image were corrected by using ExploreDTI (www.exploredti.com)[1].

2. The corrected FA/hFA image of each subject was transformed to the MNI152-T1-2mm template by using FMRIB's FLIRT and Non-Linear Registration Tools (FNIRT)[2]. Spline coefficients $\varphi_1 \dots \varphi_N$ were obtained between the FA/hFA image and MNI152-T1-2mm template. The FA/hFA images in MNI space (T1-MNI), $S_1 \dots S_N$, were obtained. N is the number of subjects.

3. S_i (i=1…N) was non-linearly transformed to S_j (j=1…N, j≠i) and the transformation, T_{is} (s=1…N-1), was obtained between S_i and S_j. The mean of the deformation fields of S_i to

all other images is computed as:

$$\Phi_i = \frac{1}{N-1}\sum_{i=1}^{N-1} T_{is} \quad (i = 1 \ldots N, s = 1 \ldots \mathrm{N}-1) \tag{2}$$

4. Keep repeating the above step until you obtain all of the means of the deformation fields of each subject image to all other images. Finally, the average deformation field of each subject, $\Phi_1 \ldots \Phi_N$, was obtained.

5. The consecutive application of the transformations $\varphi_i \circ \phi_i$ (i=1…N) was constructed based on the converwarp command, which is an FSL tool for combining multiple transforms into one. The composite transformation $\varphi_i \circ \phi_i$ was defined as the final deformation field in the following analysis.

Similarity metrics of inter-subject spatial normalization in actually measured data sets

The overlap of eigenvalue-eigenvector pairs (OVL) represents the rate of orientational information preservation during image normalization, which is calculated by using [3]:

$$\mathrm{OVL} = \frac{\sum_{j=1}^{3}\left(\lambda_j \lambda_j' \cdot \left(\varepsilon_j \cdot \varepsilon_j'\right)^2\right)}{\sum_{j=1}^{3} \lambda_j \lambda_j'} \tag{3}$$

where ε_j, λ_j, and ε_j', λ_j' are the eigenvalue-eigenvector pairs that were derived from the two DT images [3]. N is the number of subjects.

The Euclidean distance of the tensors and *the Euclidean distance of the deviatoric tensors* evaluate the space distance of the two tensors at each voxel, which were calculated by using:

$$\mathrm{DTED} = \sqrt{trace((D_1 - D_2)^2)} \tag{4}$$

$$\text{DVED} = \sqrt{trace\left(\left((D_1 - [trace(D_1/3)] * I) - (D_2 - [trace(D_2/3)] * I)\right)^2\right)} \quad (5)$$

where D_1 and D_2 are two diffusion tensors from two normalized data sets [4]. The average DTED and DVED of each subject image to all other images were calculated for each voxel[5].

The angle of primary eigenvectors reflects the angle of the direction of the information of co-registered tensors from different datasets, which was estimated by using:

$$\text{AI} = \cos^{-1}\left(\frac{|e_i \cdot e_i^+|}{\|e_i\| \cdot \|e_i^+\|}\right) \qquad i = 1,2,3 \quad (6)$$

where e_i and e_i^+ are the components of the primary eigenvector from the two tensors, and the mean AI of each subject image to all other images was calculated for each voxel[5,6].

The coherence of primary eigenvectors reflects the level of similarity of all of the co-registered tensors, was transformed based on different feature images, and was estimated by using:

$$\text{COH} = 1 - \sqrt{\frac{\beta_2 + \beta_3}{2\beta_1}} \quad (7)$$

where β_1, β_2 and β_3 are the first, second and third eigenvalues of the average dyadic tensor which is estimated as follows:

$$\langle \varepsilon_1 \varepsilon_1^T \rangle = \left\langle \begin{bmatrix} \varepsilon_{1x}^2 & \varepsilon_{1x}\varepsilon_{1y} & \varepsilon_{1x}\varepsilon_{1z} \\ \varepsilon_{1x}\varepsilon_{1y} & \varepsilon_{1y}^2 & \varepsilon_{1y}\varepsilon_{1z} \\ \varepsilon_{1x}\varepsilon_{1z} & \varepsilon_{1y}\varepsilon_{1z} & \varepsilon_{1z}^2 \end{bmatrix} \right\rangle = \frac{\sum_{i=1}^{N} \varepsilon_1^i \varepsilon_1^{i\,T}}{N} \quad (8)$$

where ε_1^i is the primary eigenvector of the ith subject, ε_{1x}, ε_{1y} and ε_{1z} are the three elements of the primary eigenvector, and N is the number of subjects[3,5,6].

The cross-correlation of FA values assesses the correlation of individual datasets and the DT template in the FA value was estimated by using:

$$corr_{FA}(j) = \frac{1}{N}\sum_{s=1}^{N}\frac{\sum_{x_i} FA_s\,(x_i)FA'(x_i)}{\left(\sum_{x_i} FA_s(x_i)\,FA_s(x_i)\sum_{x_i} FA'\,(x_i)FA'(x_i)\right)^{1/2}} \qquad (9)$$

where $corr_{FA}(j)$ is the $corr_{FA}$ of the jth subject. $\mathrm{FA}'(x_i)$ and $\mathrm{FA}_s(x_i)$ represent the FA value of voxel x_i from the jth subject and s^{th} subject respectively[5].

The calculation of the hFA image

As mentioned in Goodlett et al. (2009)[7]: the σ value for the kernel is chosen empirically to be proportional to the size of white matter structures in the brain. For example, a smaller value is used for neonates than for adults. The σ value is used to extract a relevant data set into feature space according to geometrical consideration. The σ value is a nearest-neighbor classifier in some sense. What the σ value does is that it represents each point with the distance to all of the other points in the dataset. In other words, the σ value adjusts to the local shape in a region, similar to how the nearest neighbor locally adjusts the boundary by looking at the distance to other points in the local region. Hence, the σ value was different based on different sizes of the image. In our manuscript, the σ value is 2.

1 Leemans, A., Jeurissen, B., Sijbers, J. & Jones, D. ExploreDTI: a graphical toolbox for processing, analyzing, and visualizing diffusion MR data. *17th Annual Meeting of Intl Soc Mag Reson Med* **209**, 3537 (2009).

2 Jenkinson, M., Beckmann, C. F., Behrens, T. E., Woolrich, M. W. & Smith, S. M. Fsl. *NeuroImage* **62**, 782-790, doi:10.1016/j.neuroimage.2011.09.015 (2012).

3 Basser, P. J. & Pajevic, S. Statistical artifacts in diffusion tensor MRI (DT-MRI) caused by background noise. *Magn Reson Med* **44**, 41-50 (2000).

4 Alexander, D. C. & Gee, J. C. Elastic matching of diffusion tensor images. *Comput Vis Image Und* **77**, 233-250, doi:DOI 10.1006/cviu.1999.0817 (2000).

5 Zhang, S., Peng, H., Dawe, R. J. & Arfanakis, K. Enhanced ICBM diffusion tensor template of the human brain. *NeuroImage* **54**, 974-984, doi:10.1016/j.neuroimage.2010.09.008 (2011).

6 Jones, D. K. *et al.* Spatial normalization and averaging of diffusion tensor MRI data sets. *NeuroImage* **17**, 592-617 (2002).

7 Goodlett, C. B., Fletcher, P. T., Gilmore, J. H. & Gerig, G. Group analysis of DTI fiber tract statistics with application to neurodevelopment. *NeuroImage* **45**, S133-S142, doi:10.1016/j.neuroimage.2008.10.060 (2009).

Supplementary Figure

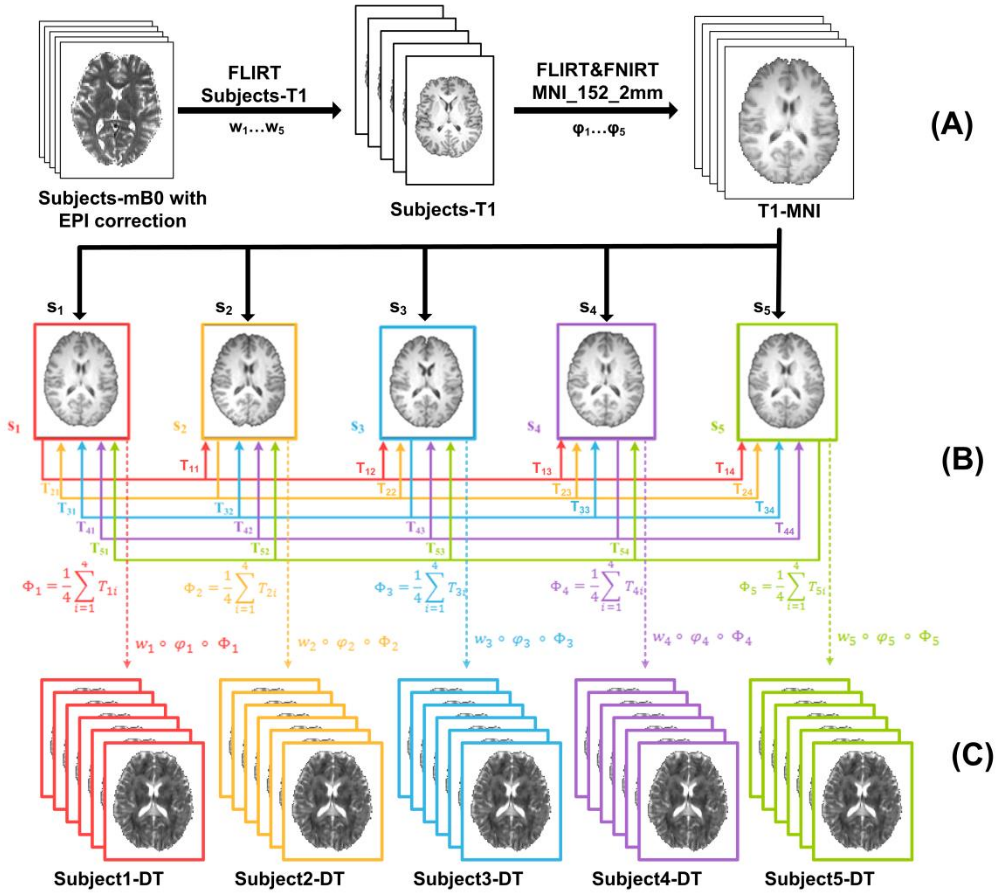

Figure S1. The schematic overview of the deformation field calculation when the T1 weighed image was selected as a feature image (in this section, we presented five data sets as an example).

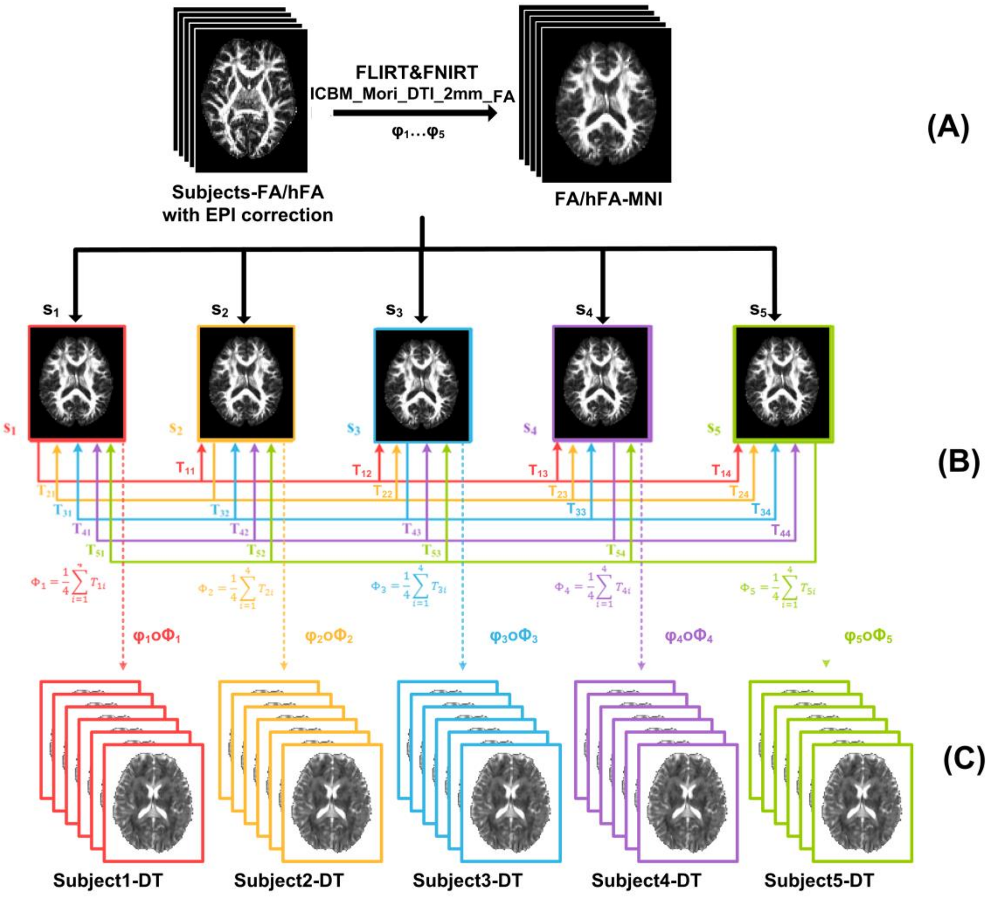

Figure S2. The schematic overview of the deformation field calculation when the FA/hFA image was selected as a feature image (in this section, we presented five data sets as an example).
